# Validated LC–MS/MS Methods for Quantification of AC02 and Porcine ACTH_1–39_ in Human Plasma: Application to a Phase I Study

**DOI:** 10.3390/ph19091397

**Published:** 2026-09-03

**Authors:** Shunbo Zhao, Bingda Wu, Hui Shen, Qi Zhou, Chang Shu, Li Ding

**Affiliations:** 1 Department of Pharmaceutical Analysis, China Pharmaceutical University, Nanjing 211198, China; zsb261902@163.com (S.Z.); wubingda0824@163.com (B.W.); 2 Nanjing Clinical Tech Laboratories Inc., Nanjing 211100, China; shenhui@klt-pharm.com (H.S.); 15255002101@163.com (Q.Z.); 3 Nanjing Jiening Pharmaceutical Technology, Nanjing 211100, China

**Keywords:** AC02 peptide, adrenocorticotropic hormone, pharmacokinetics, micro-solid-phase extraction, LC–MS/MS method

## Abstract

**Background/Objectives**: AC02 is a novel 39-amino-acid adrenocorticotropic hormone (ACTH) analogue designed for the treatment of infantile spasms. To support its clinical study, in which porcine ACTH_1–39_ served as the positive-control drug, reliable methods for the determination of AC02 and porcine ACTH_1–39_ in human plasma were required. Reported analytical methods for ACTH analogues are mainly immunoassays, which are easily affected by cross-reaction and the hook effect, necessitating a more selective analytical approach. **Methods**: Two LC-MS/MS methods were developed for the determination of ACTH analogues AC02 and porcine ACTH_1–39_ in human plasma. Human ACTH_1–39_ was included as a selectivity marker to confirm that endogenous ACTH does not interfere with the quantification of AC02. Based on the distinct concentration requirements and matrix challenges, two sample-preparation procedures were established: micro-solid-phase extraction coupled with protein precipitation for porcine ACTH_1–39_ (LLOQ 0.100 ng/mL), and acid-mediated protein precipitation for AC02 (LLOQ 0.500 ng/mL). The [M+6H]^6+^ ions were selected as precursor ions, and the corresponding 5^+^ fragment ions, formed by loss of the C-terminal phenylalanine, were used for MRM detection. **Results**: Despite a mass difference of only 0.98 Da between AC02 and human ACTH_1–39_, which are indistinguishable by mass spectrometry, baseline chromatographic separation was achieved. Both methods were fully validated in accordance with current bioanalytical guidelines. **Conclusions**: The validated methods were successfully applied to the phase I clinical study of AC02 and porcine ACTH_1–39_, enabling reliable quantification of the drug candidate and its active comparator, porcine ACTH_1–39_.

## 1. Introduction

Adrenocorticotropic hormone (ACTH) is a 39-amino-acid peptide secreted by the anterior pituitary gland and serves as a first-line therapy drug for the treatment of infantile spasms, exerting its effects through melanocortin-2 receptor activation and subsequent stimulation of cortisol release [1,2,3,4]. The high cost and limited availability of natural ACTH formulations have driven the development of synthetic analogues. Currently, two main ACTH formulations are used clinically: natural porcine ACTH_1–39_ (e.g., Repository Corticotropin Injection in the US and Corticotropin for Injection in China), extracted from porcine pituitary, and synthetic ACTH_1–24_ (tetracosactide), comprising the N-terminal 24 amino acids, which is used in Europe and other regions [5,6]. The natural product contains the full 39-amino-acid sequence but suffers from batch-to-batch variability and supply chain limitations, while the synthetic 1–24 fragment has a shorter half-life and different pharmacokinetic properties. AC02 (U.S. Patent No.: US11419919B) is a novel 39-amino-acid synthetic peptide with an Asn^25^ → Asp^25^ substitution of human ACTH_1–39_ and is currently under investigation in a phase I clinical trial. This single amino acid substitution was designed to eliminate a deamidation site at Asn25, thereby improving chemical stability, while preserving the full-length 39-amino-acid sequence for optimal receptor interaction. AC02 is a fully synthetic peptide with defined structure and high purity (>97%), whereas porcine ACTH_1–39_ is derived from porcine pituitary extracts and may contain other ACTH-related peptides or degradation products, as reported in the literature [7]. However, the direct impact of these compositional differences on clinical outcomes has not been systematically evaluated. Reliable quantification of the exogenous therapeutic peptide AC02 and the positive-control drug porcine ACTH_1–39_ in human plasma is crucial for dose optimization and pharmacokinetic assessment in ongoing clinical trials.

At present, reported methods for the determination of ACTH in human plasma are mainly immunoassays [8,9,10]. Inadequate antibody specificity, cross-reaction, limited dynamic range, and inter-kit variability are common limitations of these immunoassays, which can cause inter-laboratory disparities and confuse clinical interpretations [11,12,13,14,15,16]. Immunoassays are also vulnerable to antigen excess and the hook effect at supraphysiological levels of therapeutic dosage, which could compromise accuracy [17,18,19]. In contrast, liquid chromatography–tandem mass spectrometry (LC-MS/MS) has become a gold standard technique for quantifying peptides in human plasma due to its excellent specificity, wide dynamic range, and multiplexing capabilities [20,21,22,23,24,25]. However, to date, no fully validated LC-MS/MS method has been reported for the determination of intact ACTH_1–39_ and its structural analogues in human plasma. Most existing LC-MS/MS methods have focused on the detection of the ACTH_1–24_ fragment or have employed immunoaffinity capture techniques, which are susceptible to cross-reaction and do not provide direct separation and quantification of the full-length 39-amino-acid peptide. For instance, Shi et al. described an immunoenrichment LC-MS/MS method for intact ACTH_1–39_ quantification using 500 μL of plasma; however, this method was not fully validated according to current bioanalytical guidelines and was primarily used to evaluate measurement discrepancies between different immunoassays [26]. Additionally, previously reported chromatographic conditions were developed for Synacthen (ACTH_1–24_) and related derivatives, offering limited resolution for intact ACTH_1–39_ [27,28].

**Figure 1:**
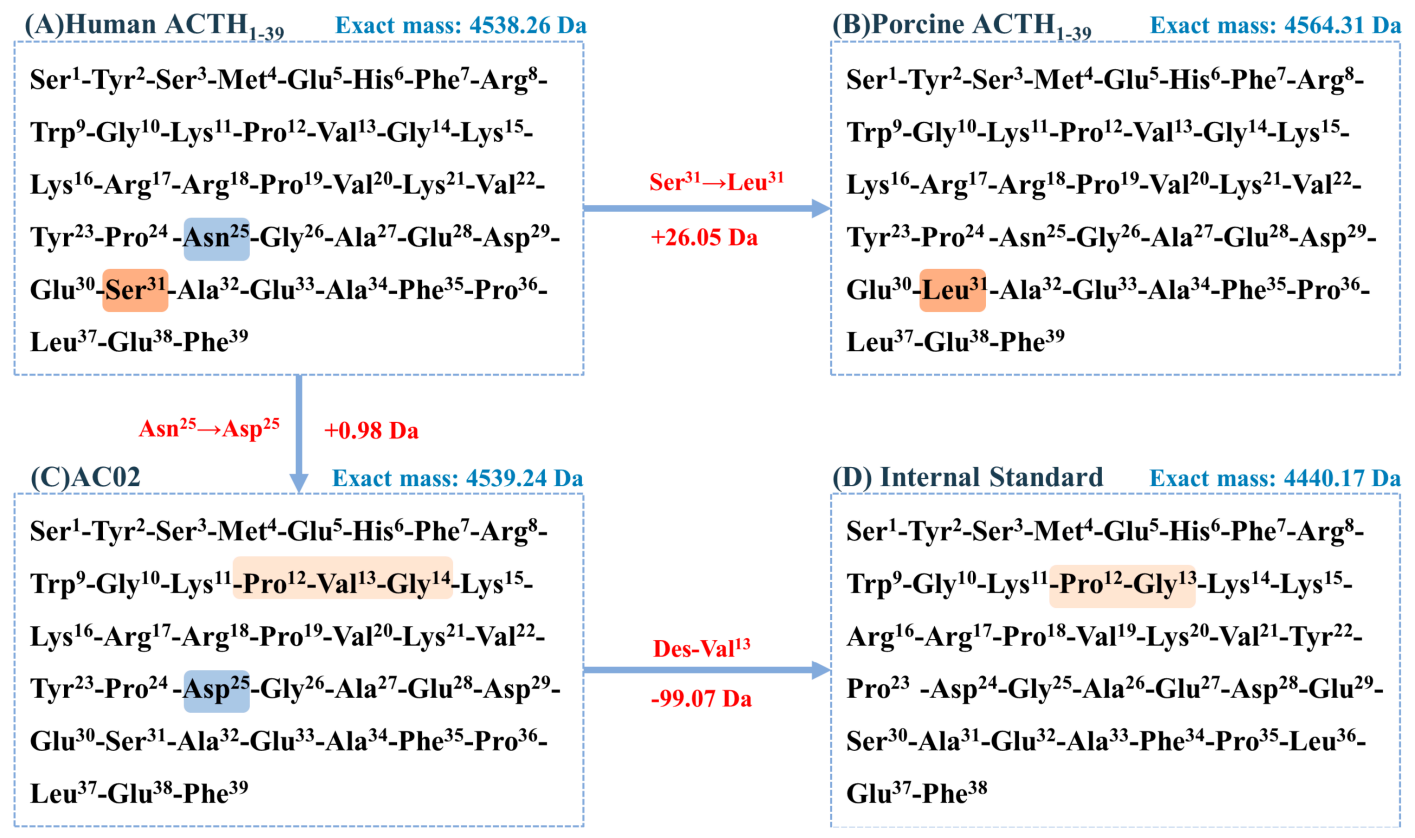
Amino acid sequences of the three ACTH isoforms and the internal standard Des-Val^13^. (**A**) Human ACTH_1–39_ (4538.26 Da). (**B**) AC02 (Asn^25^ → Asp^25^, +0.98 Da). (**C**) Porcine ACTH_1–39_ (Ser^31^ → Leu^31^, +26.05 Da). (**D**) IS Des-Val^13^ (lacks Val^13^, 4440.17 Da). Color coding: blue indicates Asn^25^ → Asp^25^ (AC02), orange indicates Ser^31^ → Leu^31^ (porcine), and light orange indicates the Val^13^ deletion (internal standard).

Given these limitations, the development of a fully validated LC-MS/MS method for simultaneous quantification of endogenous and exogenous intact ACTH_1–39_ analogues is warranted. A major analytical challenge lies in achieving effective chromatographic separation of peptides with minimal structural differences. Porcine ACTH_1–39_ and AC02 are examples of this challenge. Porcine ACTH_1–39_ has an exact mass 26.05 Da higher than that of human ACTH_1–39_, resulting from the substitution of Leu^31^ (porcine) for Ser^31^ (human). In contrast, AC02 differs from human ACTH_1–39_ by only 0.98 Da, due to the Asn^25^ (human) → Asp^25^ (AC02) substitution. Given their tiny mass difference of 0.98 Da, AC02 and human ACTH_1–39_ are almost indistinguishable by mass spectrometry alone. While the Asn-to-Asp substitution introduces a polarity change that could theoretically facilitate chromatographic separation on a reversed-phase column, in practice, the influence of a single amino acid change on overall retention behavior is often subtle for a 39-amino-acid peptide. The local polarity difference may be masked by the length of the peptide chain and the formation of specific spatial conformations, making baseline separation challenging. However, the spatial conformations of these peptides in the mobile phase might be influenced by variations in mobile phase composition, such as modifying gradient profiles or the pH values of the mobile phase. These alterations in mobile phase might enable their chromatographic separation by amplifying the minute variations in their spatial conformations [29,30,31]. The reason is that a relatively more extended peptide structure may exhibit stronger interactions with the bonded-phase molecular chains in the column, resulting in increased retention [32]. The present study aimed to address this challenge by developing a selective LC-MS/MS method capable of distinguishing and quantifying AC02, human ACTH_1–39_ and porcine ACTH_1–39_ (Figure 1). The developed methods were intended to support the pharmacokinetic evaluation of AC02 and porcine ACTH_1–39_ in a phase I clinical study.

## 2. Results

### 2.1. Mass Characteristics and MRM

**Figure 2:**
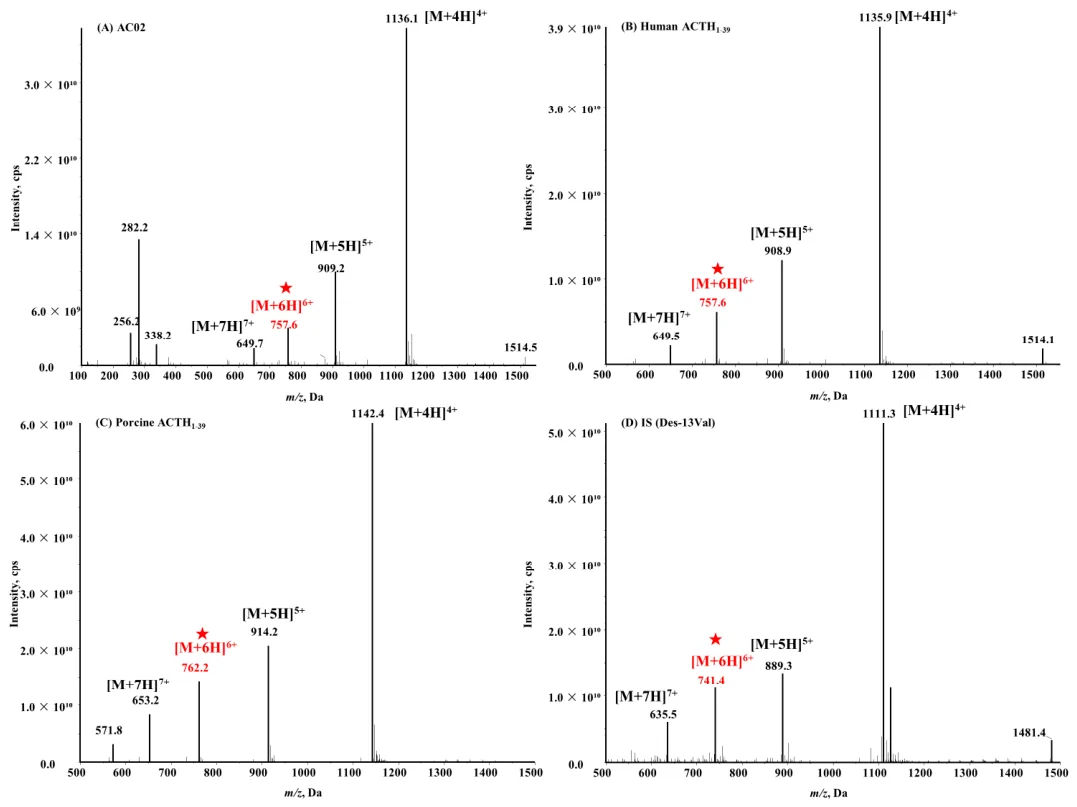
The Q1 full-scan mass spectra of AC02 (**A**), human ACTH_1–39_ (**B**), porcine ACTH_1–39_ (**C**) and the IS (**D**). Four peptides exhibited protonated ions with 4^+^ to 7^+^ charge states. The ions of [M+6H]^6+^ for each peptide were selected as the precursor ions for subsequent secondary fragmentation. The asterisks indicate the parent ions selected for MRM transition.

**Figure 3:**
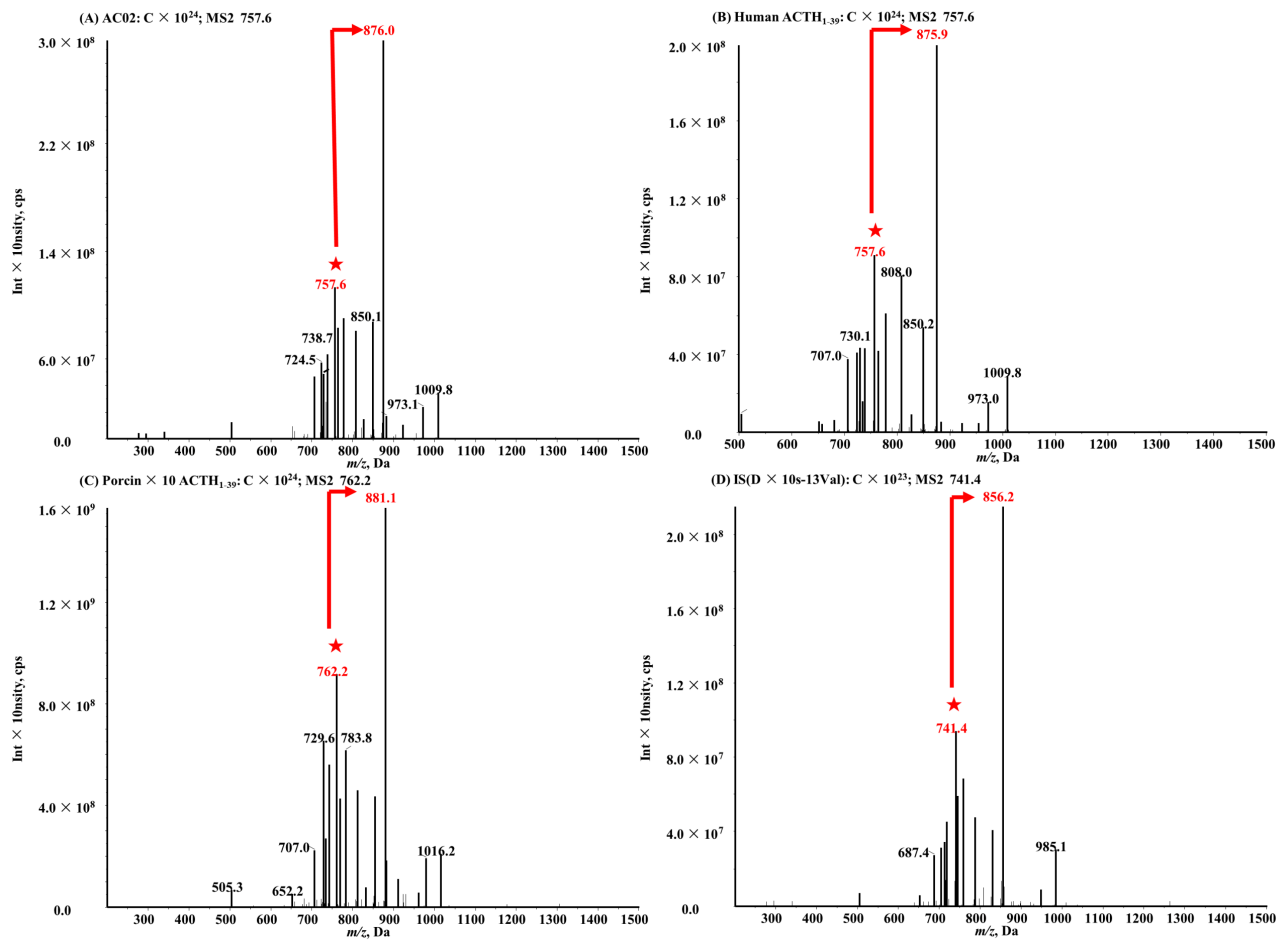
The product ion mass spectra of the [M+6H]^6+^ precursor ions for AC02 (**A**), human ACTH_1–39_ (**B**), porcine ACTH_1–39_ (**C**), and the IS (**D**). The asterisks indicate the parent ions selected for MRM transition, while the arrows denote the corresponding product ions.

In the Q1 full-scan mass spectra (Figure 2), all four peptides showed the protonated ions of different charge states [M+4H]^4+^, [M+5H]^5+^, [M+6H]^6+^ and [M+7H]^7+^ in positive-ion mode, in which the intensity values of the [M+4H]^4+^ ions were the highest for all the peptides. However, in the MS/MS spectra (Figure 3), the [M+6H]^6+^ ions of each peptide produced the most stable and highest product ion signals, which were selected as the precursor ions for their multiple reaction monitoring (MRM) transitions. A comparison of MRM signal intensities for the different charge states (4^+^ to 7^+^) for each peptide is provided in Supplementary Table S1. Although the [M+4H]^4+^ ions consistently showed the highest intensity in Q1 full scan across all four peptides, the [M+6H]^6+^ ions were selected for MRM detection because they yielded the most stable and reproducible product ion signals in MS/MS. In addition, for porcine ACTH_1–39_—which had the most stringent LLOQ requirement (0.100 ng/mL)—the [M+6H]^6+^ ion also exhibited the highest intensity among all charge states tested. To maintain consistency across all analytes, the [M+6H]^6+^ charge state was adopted for all four peptides.

### 2.2. Chromatographic Separation

**Figure 4:**
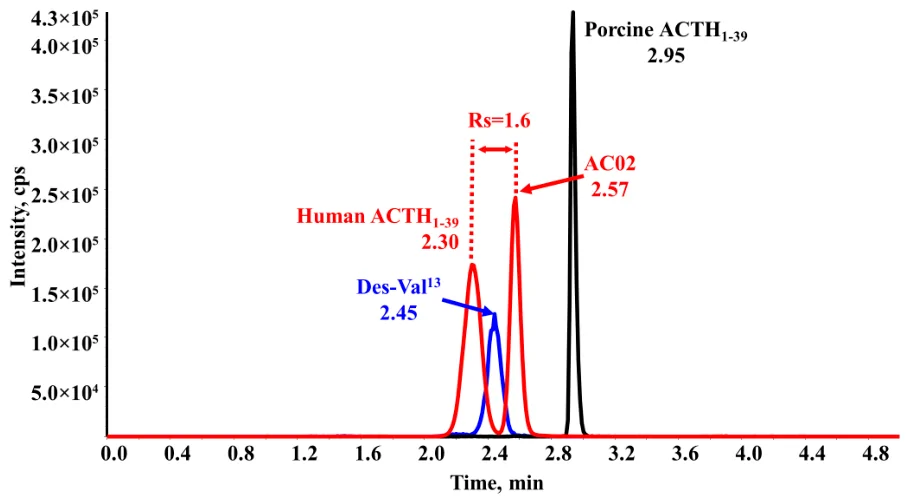
Representative chromatogram showing the separation of human ACTH_1–39_, the internal standard, AC02, and porcine ACTH_1–39_. The red trace indicates the MRM chromatograms of AC02 and human ACTH_1–39_, the black trace indicates the porcine ACTH_1–39_ and the blue trace indicates the IS. Baseline separation between AC02 and endogenous human ACTH_1–39_ (Δm = 0.98 Da, rendering them indistinguishable by mass spectrometry) was achieved with a resolution (Rs) of 1.6 and symmetrical peak profiles. Column: ACQUITY UPLC^®^ CSH™ C18 (50 × 2.1 mm, 1.7 µm). Elution order: human ACTH_1–39_ → IS → AC02 → porcine ACTH_1–39_.

The most significant analytical challenge in this study was to achieve baseline separation between AC02 and endogenous human ACTH_1–39_. These two peptides differ by only 0.98 Da in exact mass. This mass difference is further diminished by the mass averaging effect arising from the multiple charging of ions formed during electrospray ionization, which means that AC02 and human ACTH_1–39_ are almost indistinguishable by mass spectrometry. Moreover, the high sequence homology (97%) between the two peptides poses an additional obstacle to chromatographic separation. However, the tertiary structures of these peptides in the mobile phase might be influenced by the variation in the composition of mobile phase, such as modifying the gradient profiles or the pH values of the mobile phase, which can modulate their interactions with the stationary phase and thereby enable chromatographic resolution [33,34]. Several reversed-phase columns with different stationary phases were screened. The ACQUITY UPLC^®^ CSH™ C18 column (50 × 2.1 mm, 1.7 μm; Waters) (Milford, MA, USA) provided satisfactory performance, achieving symmetrical peak shapes and resolutions (Rs) of 1.6 between AC02 and human ACTH_1–39_ (Figure 4). Mobile phase acidity was found to have a marked effect on separation; the optimal combination was 0.2% formic acid in water (A) and 0.2% formic acid in acetonitrile (B). To further improve the resolution between the closely eluting peptides of AC02 and human ACTH_1–39_, the following shallow gradient was employed for separation optimization and method development: 20% B from 0 to 1.5 min, followed by a linear increase from 20% to 26% B over 1.5 to 3.5 min. However, subsequent analysis of baseline samples from the positive-control group revealed that endogenous human ACTH_1–39_ levels remained below 0.100 ng/mL in all subjects, with interference at the retention time of AC02 below 20% of the LLOQ peak area. This allowed the use of an adjusted gradient for routine clinical sample analysis, as described in Section 4.2.

### 2.3. Sample Preparation Optimization

For porcine ACTH_1–39_, the required LLOQ was 0.100 ng/mL. A single protein precipitation procedure was not sufficient for the clean-up and enrichment of the plasma samples, due to significant matrix effects and poor extraction recovery resulting from the co-precipitation of the ACTH peptides with the endogenous plasma proteins. We compared the performance of simple protein precipitation alone versus the combined protein precipitation/μSPE workflow for porcine ACTH_1–39_. With protein precipitation alone, the recovery was only 52–61%, and the signal-to-noise ratio at the LLOQ was < 5, which was inadequate for reliable quantification at 0.100 ng/mL. In contrast, the combined protein precipitation/μSPE procedure yielded significantly improved performance: recovery of 80–90%, an IS-normalized matrix factor of 0.9–1, and a signal-to-noise ratio >10 at the LLOQ. Therefore, a hybrid sample preparation strategy combining protein precipitation with micro-solid-phase extraction (μSPE) was employed. In the protein precipitation step, different types of precipitants were investigated, including methanol, acetonitrile and their various combinations with formic acid and zinc sulfate. A mixture of water and acetonitrile (1:4, *v*/*v*) was proven as the optimal precipitant. For the subsequent μSPE clean-up, three types of μElution plates (Oasis^®^ BEH, Oasis^®^ MCX and Oasis^®^ MAX) (Waters, Milford, MA, USA) were tried. Finally, the Oasis^®^ MAX, an anion exchange μSPE plate, was selected.

For AC02, a single protein precipitation procedure was employed. The plasma samples were acidified with 2% formic acid prior to precipitation. A mixture of methanol/acetonitrile (1:1, *v*/*v*) was selected as the precipitation solvent to improve matrix effect and recovery. The method development for AC02 began with protein precipitation optimization. Since the established protein precipitation method already provided sufficient sensitivity (LLOQ 0.500 ng/mL) with acceptable recovery (78.9–82.7%) and matrix effects (RE% −1.3 to 0.7%), the µSPE workflow was not further pursued. Nonspecific adsorption is a common phenomenon in peptide bioanalysis [35]. To avoid nonspecific binding, stock/working solutions were prepared in a stabilizing mixture that contained HSA [36]. Additionally, the tubes of low-binding polypropylene materials were employed throughout the sample preparation process.

### 2.4. Method Validation Results

The method demonstrated excellent selectivity, with no endogenous interference observed at the retention times of any analyte or the internal standard in plasma samples from six individual plasma lots (Figure S1) and hemolyzed and lipemic samples (Figure S2). In the clinical study, baseline samples from all 10 participants in the positive-control group were analyzed to evaluate the potential interference of endogenous human ACTH_1–39_. The results showed that endogenous human ACTH_1–39_ levels remained below 100 pg/mL in all participants, and the interference at the retention time of AC02 was below 20% of the LLOQ peak area, confirming the absence of significant physiological interference during the study. Calibration curves for both analytes exhibited good linearity over their respective ranges (0.500–200 ng/mL for AC02 and 0.100–10.0 ng/mL for porcine ACTH), with correlation coefficients (*R*^2^) > 0.99 using a 1/*x*^2^ weighted linear regression model. The back-calculated accuracy for each calibration standard across three independent runs is provided in Supplementary Table S2 (AC02) and Table S3 (porcine ACTH_1–39_). Intra- and inter-run accuracy and precision met all acceptance criteria across all QC levels; results are summarized in Supplementary Table S4. To assess ruggedness, the developed method was further validated by using different analysts, columns and instrumentations. All results remained within the predefined acceptance criteria. The LLOQs (0.500 ng/mL for AC02; 0.100 ng/mL for porcine ACTH_1–39_) were suitable to characterize the complete plasma concentration–time profiles in the clinical study. Mean absolute recovery ranged from 79% to 90% for both analytes across QC levels (Table S5). The matrix factor ranged from −4.3 to 0.7 (RE %) with an RSD < 15% across six different individual plasma lots, indicating negligible ion suppression/enhancement and consistent IS performance. The suitability of Des-Val^13^ as the internal standard was confirmed by the absence of mutual interference at the respective retention times, and comparable recovery and matrix effects to the analytes, with IS-normalized accuracy and precision meeting all acceptance criteria. Comprehensive stability testing (Table S6) confirmed that both analytes were stable in plasma under all relevant conditions: short-term on wet ice (~20 h), up to five freeze–thaw cycles, long-term storage at −20 °C and −70 °C for at least 61 days, and in the processed extract in the autosampler (8 °C) for up to 52 h. The instability of ACTH in whole blood/plasma at room temperature, attributed to potential enzyme-mediated degradation, underscores the importance of the defined sample handling protocol (immediate ice-bath placement and prompt centrifugation).

### 2.5. Clinical Application

**Figure 5:**
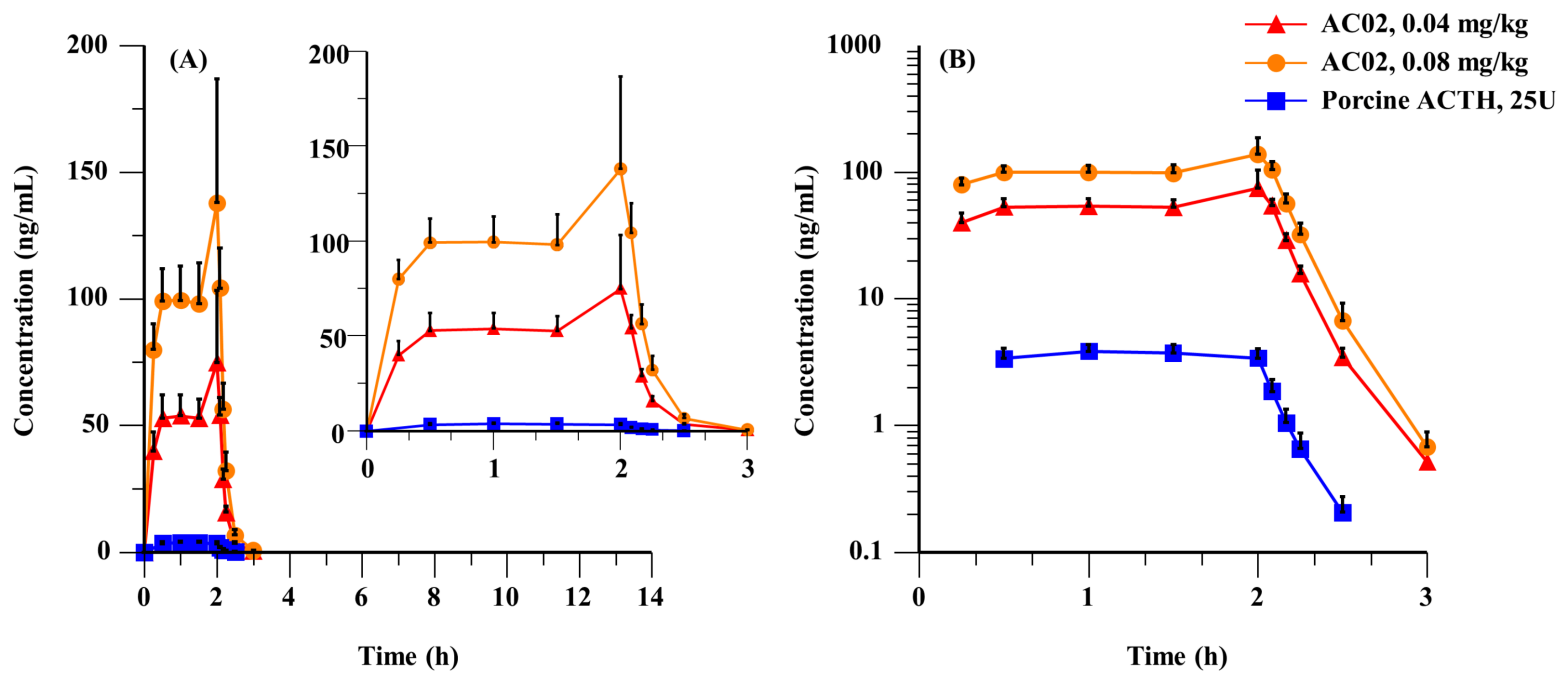
Mean plasma concentration–time profiles of AC02 and porcine ACTH_1–39_ in healthy volunteers (*n* = 10). (**A**) Linear-scale plot showing the complete pharmacokinetic time course (0–14 h). Inset highlights the first 3 h post-dose, after which both analyte concentrations declined below the LLOQ. (**B**) Semi-logarithmic plot of the initial 3 h post-dose.

The validated methods were successfully applied to a phase I clinical trial, providing clear pharmacokinetic characterization of both AC02 and porcine ACTH_1–39_. As illustrated in Figure 5, the plasma concentration profiles show different exposure patterns for the two drugs. The short elimination half-lives (*t*_1/2_) of AC02 and porcine ACTH_1–39_ (Table S7) are similar to those reported for other ACTH analogues, supporting their expected rapid clearance in vivo. Notably, AC02 (0.04 mg/kg) demonstrated approximately 17-fold higher systemic exposure compared to porcine ACTH_1–39_ (25 U). However, these findings are derived from a phase 1 study with a limited sample size (*n* = 10 per group), and the observed variability (CV% ranging from 9.1% to 34.5% for AC02 and from 14.7% to 15.3% for porcine ACTH_1–39_ across PK parameters) should be considered when interpreting the exposure difference.

## 3. Discussion

The initial objective was to develop an LC-MS/MS method for the simultaneous quantification of AC02, human ACTH_1–39_ and porcine ACTH_1–39_. However, the baseline concentration of human ACTH_1–39_ was not detected in the plasma samples from the positive-control group, indicating that monitoring endogenous human ACTH_1–39_ was not necessary. As plasma samples containing AC02 or porcine ACTH_1–39_ were derived from separate study groups, two independent quantitative methods were developed and validated for each analyte. Two distinct sample-preparation workflows were developed to address the different sensitivity requirements and matrix challenges of the two analytes. For AC02, a simple acid-mediated protein precipitation method was sufficient, as the higher LLOQ (0.500 ng/mL) allowed adequate sensitivity without additional cleanup. For porcine ACTH_1–39_, the more demanding LLOQ (0.100 ng/mL) necessitated a combined protein precipitation and μSPE approach. The μSPE step effectively removed phospholipids and other matrix components that caused significant ion suppression, enabling reliable quantification at the sub-ng/mL level. A comparative summary of the method performance is presented in Table 1. Although the porcine ACTH_1–39_ method is more labor-intensive, the improved sensitivity and selectivity were necessary to fully characterize the lower exposure levels of the positive-control drug in the clinical study. This strategy demonstrates that the choice of sample preparation should be guided by the specific sensitivity and matrix requirements of each analyte.

The recovery of porcine ACTH_1–39_ (83.8–90.5%) and AC02 (78.9–82.7%) is modest despite the anti-adsorption measures implemented (low-binding tubes, HSA-containing solutions, and acidified diluents). This is primarily attributed to the sample preparation strategy: For porcine ACTH_1–39_, the μSPE workflow inherently involves multiple transfer, washing, and elution steps, each contributing to cumulative sample loss. For AC02, the moderate recovery may be due to co-precipitation with endogenous proteins during protein precipitation. Importantly, the recovery is highly reproducible (RSD ≤ 4.1%), and the IS-normalized recovery is well within acceptable limits. The consistency of recovery across concentrations confirms that the losses are systematic rather than random and are effectively compensated by the internal standard. Therefore, the moderate absolute recovery does not affect the accuracy or precision of the method.

From the MS/MS spectra, the characteristic product ions of each peptide exhibited mass-to-charge ratio (*m*/*z*) values higher than those of their corresponding precursor ions. Based on the observed fragmentation pattern, those product ions were identified as 5+ charged fragments generated by cleavage at the C-terminal of these ACTH analogues, specifically via the loss of phenylalanine at position 39 (Phe^39^) in their peptide sequences. The ionization forms, charge state distributions and the characteristic de-phenylalanine fragmentation behavior of these ACTH analogues are summarized in Table 2. Isotopically labeled internal standards often suffer from poor selectivity due to the mass averaging effect caused by multiple charging during electrospray ionization of peptides [37]. Therefore, a structurally similar peptide (AC02 lacking valine at position 13) was employed as the internal standard instead of a stable isotope-labeled analogue.

Under the optimized chromatographic conditions, baseline separation of AC02, human ACTH_1–39_, and porcine ACTH_1–39_ was achieved. The observed elution order (human ACTH_1–39_ < AC02 < porcine ACTH_1–39_) was consistent across different column types. This separation behavior may be attributed to variations in peptide conformation and tertiary structure size in the mobile phase, which may be influenced by the composition of the mobile phase. While direct structural evidence is lacking, the following interpretation is consistent with the observed chromatographic behavior. The difference in the structure between AC02 and human ACTH_1–39_ is that the Asn^25^ (human ACTH_1–39_) is substituted by Asp^25^ (AC02), where the amide group is substituted by a carboxyl group. It is plausible that, in the hydrophilic mobile phase, the amide group in human ACTH_1–39_ favors a more compact and stable tertiary structure, which could result in weaker interaction with the stationary phase and consequently a shorter retention time. In contrast, the carboxyl group in AC02 may enhance its interaction with the hydrophilic mobile phase, potentially leading to a more extended conformation, stronger interaction with the stationary phase, and thus longer retention—enabling complete separation from human ACTH. For porcine ACTH_1–39_, the substitution of Ser^31^ (human ACTH_1–39_) with Leu^31^ (porcine ACTH_1–39_) replaces the polar hydroxyl group of serine with the non-polar, highly hydrophobic isobutyl group of leucine, substantially increasing its hydrophobicity. Consequently, porcine ACTH_1–39_ exhibits the longest retention time among the three analogues on the reversed-phase column. This chromatographic strategy provides a general solution for separating peptide analogues with minor structural differences, effectively transforming a mass spectrometry-indistinguishable problem into a feasible chromatographic separation challenge. It also avoids the inherent inability of immunoassays to distinguish the endogenous ACTHs and the structurally similar peptides.

To contextualize the analytical performance of the present method, a comparison with previously reported LC-MS/MS methods for ACTH quantification is provided in Table 3. Although the LLOQ (0.100–0.500 ng/mL) is higher than that of immunocapture-based methods (~9–10 pg/mL), the method is fully validated according to ICH M10 guidelines and requires a smaller sample volume (50 μL for AC02). This makes the method particularly suitable for clinical pharmacokinetic studies, where reduced blood draw volumes improve participant comfort and facilitate repeated sampling, while the simplified workflow supports high analytical throughput.

For clinical application, AC02 is a synthetic peptide with defined structure and high purity (> 97%), whereas porcine ACTH_1–39_ is derived from porcine pituitary extracts and may contain other ACTH-related peptides or degradation products. The potential compositional differences between the synthetic and naturally derived products warrant further investigation to establish their clinical significance. It is noteworthy that AC02 (0.04 mg/kg) demonstrated approximately 17-fold higher systemic exposure compared to porcine ACTH_1–39_ (25 U). However, this difference should be interpreted with caution, as AC02 was dosed in mg/kg whereas porcine ACTH_1–39_ was dosed in Units, making direct exposure comparison inherently limited. Despite the higher AC02 exposure, the pharmacodynamic effects (free cortisol responses) were comparable between the two treatments [38]. In the positive-control group, the endogenous human ACTH_1–39_ levels remained below 0.100 ng/mL in all subjects, confirming the absence of significant physiological interference during the study. The established LLOQs of 0.100 ng/mL (porcine ACTH_1–39_) and 0.500 ng/mL (AC02) were sufficient for the full characterization of pharmacokinetic profiles, including the determination of *C*_max_, *T*_max_, *AUC* and elimination phases. The PK data presented here are derived from a phase 1 study with limited sample sizes (*n* = 10 per group). The observed variability (CV% ranging from 9.1% to 34.5% for AC02 and from 14.7% to 15.3% for porcine ACTH_1–39_ across PK parameters) should be considered when interpreting the magnitude of exposure differences.

## 4. Materials and Methods

### 4.1. Chemicals and Reagents

AC02 (Asn^25^-deamidated human ACTH_1–39_, 97.7%) and human ACTH_1–39_ (99.0%) were obtained from Nanjing Hanxin Pharmaceutical Technology Co., Ltd. (Nanjing, China). Porcine ACTH_1–39_ (97.9%) and the internal standard Des-Val^13^ (96.02%) were sourced from Synpeptide Co., Ltd. (Nanjing, China). Adrenocorticotropine for Injection (active ingredient: porcine ACTH_1–39_) was supplied by Shanghai First Biochemical Pharmaceutical Co., Ltd. (Shanghai, China). HPLC-grade methanol (MeOH), acetonitrile (ACN), and ammonium hydroxide (NH_3_·H_2_O) were obtained from Merck KGaA (Darmstadt, Germany). Formic acid (FA) and human serum albumin (HSA) were sourced from Sigma-Aldrich (St. Louis, MO, USA). Drug-free human plasma was provided by the Women & Children’s Hospital of Hunan (Changsha, China) (Ethics Approval Number: FE-GCP-LS-2024–022-T).

### 4.2. LC-MS/MS Methods

The quantification of AC02 and porcine ACTH_1–39_ was performed using a SCIEX Exion LC system coupled with a SCIEX TRIPLE QUAD™ 6500^+^ mass spectrometer (Framingham, MA, USA) equipped with an electrospray ionization (ESI) source. The chromatographic separation was achieved on an ACQUITY UPLC^®^ CSH™ C18 column (50 × 2.1 mm, 1.7 μm; Waters) (Milford, MA, USA) maintained at 45 °C. The mobile phases consisted of (A) 0.2% formic acid in water and (B) 0.2% formic acid in acetonitrile. For routine analysis of clinical samples, an adjusted gradient was employed, as the endogenous human ACTH_1–39_ was found to be below the LLOQ in all subjects. Gradient elution was performed at a constant flow rate of 0.3 mL/min as follows: 0–0.5 min, 15% B; 0.5–2.0 min, linear increase to 20% B; 2.0–3.0 min, hold at 20% B; 3.0–3.9 min, linear increase to 90% B; 3.9–4.9 min, hold at 90% B; 4.9–5.0 min, linear decrease to 15% B; 5.0–6.0 min, re-equilibrate at 15% B. The mass spectrometric detection was operated in positive electrospray ionization (ESI^+^) mode with the following multiple reaction monitoring (MRM) transitions: *m*/*z* 757.6 → 876.0 for AC02, *m*/*z* 762.2 → 881.3 for porcine ACTH_1–39_, *m*/*z* 757.6 → 875.9 for human ACTH_1–39_ and *m*/*z* 741.4 → 856.4 for the internal standard. The ion source parameters were set as follows: IonSpray voltage, 5500 V; source temperature, 450 °C; curtain gas, 40 psi; GS1, 45 psi; GS2, 45 psi. For each peptide, the collision energy (CE), declustering potential (DP), entrance potential (EP) and collision cell exit potential (CXP) were set at 24, 60, 10 and 20 V, respectively. Data acquisition and processing were conducted with Analyst 1.6.3 and Watson LIMS 7.5 SP1 software.

### 4.3. Stock and Working Solution Preparation

Stock solutions (1.00 mg/mL) of AC02, porcine ACTH_1–39_ and the internal standard Des-Val^13^ were prepared individually in a stabilizer solution composed of 40.0 mg/mL HSA in water/FA/MeOH/water (1:1:30:70, *v*/*v*/*v*/*v*) to prevent adsorptive losses. Working solutions were prepared by serial dilution in the same stabilizer solution. The internal standard working solution was prepared at 50.0 ng/mL. All solutions were stored in polypropylene tubes at −20 °C. Calibration standards and quality control samples were prepared by spiking appropriate volumes of working solutions into blank human plasma. The calibration range of AC02 was 0.500–200 ng/mL, and the concentration levels were 0.500, 1.00, 4.00, 15.0, 60.0, 120, 160, and 200 ng/mL. The calibration range of porcine ACTH_1–39_ was 0.100–10.0 ng/mL, and the concentration levels were 0.100, 0.200, 0.400, 1.20, 3.00, 6.00, 8.00, and 10.0 ng/mL. The QC samples were prepared at five concentration levels of 0.500, 1.50, 10.0, 80.0 and 150 ng/mL of AC02 and 0.100, 0.300, 1.00, 4.00 and 7.5 ng/mL of porcine ACTH_1–39_, respectively.

### 4.4. Sample Preparation

Two distinct sample preparation workflows were optimized based on the required sensitivity and analyte properties. Porcine ACTH_1–39_ was extracted using a hybrid protein precipitation and micro-solid-phase extraction (μSPE) protocol (Figure S3). Briefly, a 200 μL aliquot of plasma was subjected to protein precipitation using an optimized acetonitrile–water mixture and further processed by SPE with an Oasis^®^ MAX μElution Plate (30 μm, Waters). The preparation of AC02 samples was performed by protein precipitation. Briefly, to each well of a 96-well plate containing 50.0 µL of plasma sample, 25.0 µL of internal standard working solution was added. The plate was sealed and vortexed for 1 min; subsequently, 25.0 µL of 2% FA in water was added and vortexed for 5 min. Then, 250 µL of precipitant (methanol: acetonitrile, 1:1, *v*/*v*) was added, vortexed for 5 min, and centrifuged at 2200× *g* for 10 min at 15 °C. The supernatant (150 µL) was transferred into a new 96-well plate containing 150 µL of 2% FA in water in each well, vortexed for 2 min. The processed samples were injected into the LC-MS/MS system for analysis.

### 4.5. Method Validation

The methods were validated for selectivity, linearity, accuracy, precision, recovery, matrix effect, and stability in accordance with the International Council for Harmonisation M10 guideline [39]. Selectivity was assessed by analyzing six individual blank plasma samples, including hemolyzed and lipemic samples, to confirm the absence of endogenous interferences at the retention times of the analytes and internal standard. Linearity was evaluated by analyzing calibration curves in duplicate. A linear regression model with a weighting factor of 1/*x*^2^ was applied. Assay accuracy and precision were evaluated using QC samples at five different concentration levels. Intra-run precision was determined from six replicates analyzed in a single run, while inter-run precision was evaluated over three consecutive validation days. For all QC levels, precision (expressed as relative standard deviation, RSD) and accuracy (expressed as relative error, RE) were required to be within ±15% of the nominal concentration, except at the lower limit of quantitation (LLOQ), where a limit of ±20% was accepted. Recovery was determined by comparing the peak areas of pre-spiked QC samples (low, medium, and high concentrations; *n* = 6) with those of corresponding post-spiked pre-extracted blank plasma. The precision of recovery was required to be ≤15%. Matrix effects were evaluated using six different sources of blank matrix. For each source, low- and high-concentration QC samples (*n* = 3 per concentration) were prepared and analyzed; the resulting accuracy and precision were required to be within ±15% of the nominal concentration and ≤15% RSD, respectively. The influence of hemolyzed and lipemic matrices was also investigated. AC02 stability was evaluated under the following conditions: short-term stability at room temperature (2.4 h) and on ice (20.2 h), long-term stability at −20 °C and −70 °C (78 days), freeze–thaw stability after five cycles at −20 °C and −70 °C, and processed sample in the autosampler at 8 °C for 3 days. Porcine ACTH_1–39_ stability was similarly assessed under short-term conditions (room temperature for 2.5 h; on ice for 19.8 h), long-term storage at −20 °C and −70 °C (61 days), five freeze–thaw cycles at both temperatures, and processed sample stability in the autosampler at 8 °C for 2 days 4 h. Whole-blood stability on ice was evaluated at 0, 1, and 2 h for low- and high-concentration samples.

### 4.6. The Clinical Study

**Figure 6:**
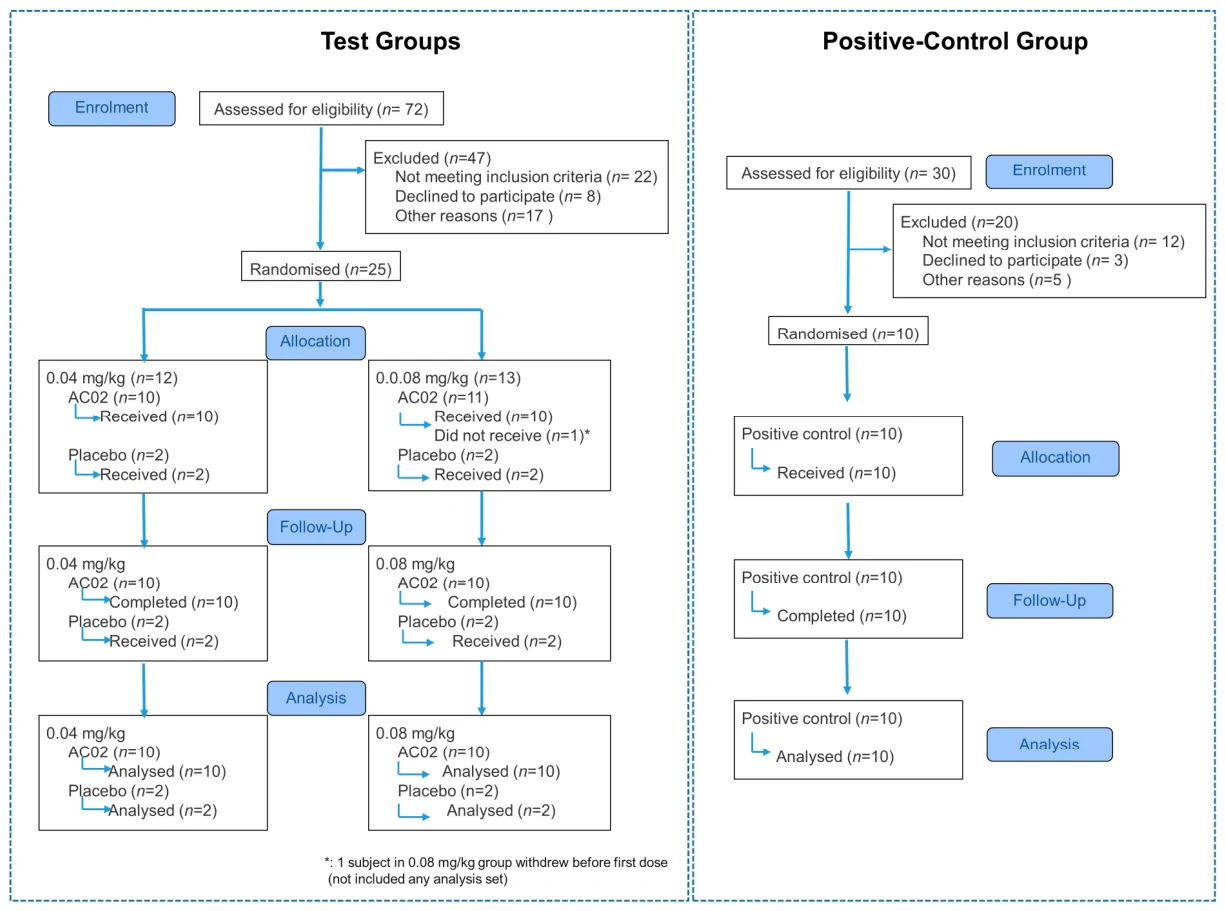
The flow diagram of the test and positive-control groups.

A randomized, double-blind, placebo- and positive-controlled phase 1 trial with 34 participants was carried out at Shandong Provincial Qianfoshan Hospital (Jinan, China) (Figure 6). The clinical trial (identifier: CTR20244455) was registered at http://www.chinadrugtrials.org.cn (accessed on 28 November 2024). The study adhered to the Declaration of Helsinki and was approved by the institutional ethics committee. In the test group, participants in each dose cohort (0.04 and 0.08 mg/kg of AC02) were randomized in a 10:2 ratio to receive either AC02 or placebo as a 2-h intravenous infusion. Another ten participants in the positive-control group received an intravenous infusion of Adrenocorticotropine for Injection (25 U) for two hours. For both the test and positive-control groups, the blood samples (4 mL at each time point) of each participant at pre-dose; 30, 60 and 90 min during infusion; immediately after infusion; and 5, 10, 15, and 30 min and 1, 2, 3, 4, 6 and 12 h post-infusion were collected into K_2_EDTA tubes. On the day before drug administration, an additional series of plasma samples without the drug taken were drawn at the same time points as those described above from the participants in the positive-control group to measure the baseline concentration levels of human ACTH_1–39_. The collected samples were placed on wet ice immediately and centrifuged at 1700× *g* for 10 min at 4 °C within 1 h, and the plasma was stored at −70 °C until analysis. Pharmacokinetic parameters were calculated with Phoenix WinNonlin 8.2 (Pharsight Corporation, Mountain View, California) by using the non-compartmental method.

## 5. Conclusions

In this study, an LC-MS/MS method was developed for the baseline chromatographic separation of the synthetic peptide AC02 and endogenous human ACTH_1–39_, which are indistinguishable by MS owing to a mere 0.98 Da mass difference. The ionization patterns, protonated ion charge states and characteristic de-phenylalanine fragmentation pathways of ACTH analogues were elucidated. The analyte-specific sample preparation procedures were employed, ensuring high recovery and sensitivity. Both methods were fully validated according to current bioanalytical guidelines and successfully applied to a phase I clinical trial, providing reliable pharmacokinetic data for AC02 and porcine ACTH_1–39_.

## Figures and Tables

**Table 1:** Comparative summary of method performance for AC02 and porcine ACTH_1–39_.

Parameter	AC02 Method	Porcine ACTH_1–39_ Method
Sample volume (μL)	50	200
Sample preparation	Acid-mediated protein precipitation	Protein precipitation + μSPE
Extraction time (min)	~30	~60
LLOQ (ng/mL)	0.500	0.100
Mean absolute recovery (%)	78.9–82.7	83.8–90.5
IS-normalized matrix factor (RE%)	−1.3 to 0.7	−4.3 to 0.7
Process efficiency (%) *	~75–80	~72–78

* Process efficiency = recovery × matrix factor.

**Table 2:** The ionization forms, protonated ion charge states and de-phenylalanine MS fragmentation characteristics of the ACTH analogues.

ACTH Analogues	AC02	Human ACTH_1–39_	Porcine ACTH_1–39_	Des-Val^13^ (IS)
Molecular weight ^1^	4542.05	4541.07	4567.15	4442.92
Exact mass ^2^	4539.24	4538.26	4564.31	4440.17
[M+4H]^4+^ (*m*/*z*)	1135.81	1135.57	1142.08	1111.04
[M+5H]^5+^ (*m*/*z*)	908.85	908.65	913.86	889.03
[M+6H]^6+^ (*m*/*z*)	757.54	757.38	761.72	741.03
[M+7H]^7+^ (*m*/*z*)	649.46	649.32	653.04	635.31
MRM transitions (*m*/*z*) ^3^	757.6 → 876.0	757.6 → 875.9	762.2 → 881.3	741.4 → 856.4
Exact mass after losing phenylalanine ^4^	4374.16	4373.18	4399.23	4275.09
[M+5H]^5+^ after losing phenylalanine (*m*/*z*)	875.83	875.64	880.85	856.02

^1^: Molecular weight refers to the average molecular mass calculated using the average isotopic masses of the constituent elements. ^2^: Exact mass refers to the monoisotopic mass calculated using the most abundant isotopic composition ^3^: MRM transitions were established by selecting the [M+6H]^6+^ ions as the precursors in Q1. Following collision-induced dissociation, the precursor ions undergo a characteristic neutral loss of the C-terminal phenylalanine (Phe, 165.08 Da), generating the corresponding 5^+^ product ions, which are monitored in Q3. The calculated *m*/*z* values for these [M+5H]^5+^ product ions are provided in the last row of the table. ^4^: Exact mass after losing phenylalanine = the exact mass of ACTH analogues − the exact mass of phenylalanine (165.08).

**Table 3:** Comparison of the current LC-MS/MS method with previously reported methods for ACTH quantification.

Parameter	Shi et al. (2019) [26]	Chaabo et al. (2011) [27]	Jones et al. (2024) [28]	AC02	Porcine ACTH_1–39_
Analyte	Intact ACTH_1–39_	ACTH_1–24_ (Synacthen)	ACTH_1–24_	AC02	Porcine ACTH_1–39_
Sample volume (μL)	500	1000	300	50	200
Sample preparation	Immunocapture + PPT	Cation exchange + SPE	Immunoaffinity	PPT	PPT + μSPE
LLOQ (pg/mL)	~9	15	10	500	100
Full ICH M10 validation	Not performed	Not performed	Not performed	Performed	Performed

## Data Availability

The original contributions presented in this study are included in the article and Supplementary Materials. Further inquiries can be directed to the corresponding authors.

## Supplementary Materials

The following supporting information can be downloaded at: https://www.mdpi.com/article/10.3390/ph19091397/s1, Figure S1: Representative MRM chromatograms of AC02 (A1: blank sample, A2: LLOQ sample of 0.500 ng/mL AC02, A3: study sample of 45.3 ng/mL) and porcine ACTH_1–39_ (B1: blank sample, B2: LLOQ sample of 0.100 ng/mL porcine ACTH_1–39_, B3: study sample of 3.55 ng/mL); Figure S2: Representative hemolyzed and lipemic matrix chromatograms of AC02 (A: lipemic sample, B: hemolyzed sample; left: AC02, right: des-val^13^) and porcine ACTH_1–39_ (C: lipemic sample, D: hemolyzed sample; left: porcine ACTH_1–39_, right: des-val^13^); Figure S3: Workflow of the sample preparation protocol for porcine ACTH_1–39_. The procedure consisted of two steps: (1) protein precipitation and (2) micro solid phase extraction (μSPE); Table S1: Selection of precursor ion charge state, Table S2: Calibration curves of AC02; Table S3: Calibration curves of porcine ACTH_1–39_; Table S4: Intra- and inter-run accuracy and precision of AC02 and porcine ACTH_1–39_; Table S5: Recovery and matrix effects of AC02 and porcine ACTH_1–39_ in plasma; Table S6: The matrix stability of AC02 and porcine ACTH_1–39_ (*n* = 3); Table S7: The pharmacokinetic parameters of AC02 and porcine ACTH_1–39_.
