# Supplementary material for: Validated LC–MS/MS Methods for Quantification of AC02 and Porcine ACTH1–39 in Human Plasma: Application to a Phase I Study
Source: Pharmaceuticals (Basel). 2026 Sep 3;19(9):1397. doi: 10.3390/ph19091397 (PMC13610943; doi:10.3390/ph19091397)
Supplement: Supplementary file 1 [file pharmaceuticals-19-01397-s001.zip › pharmaceuticals-4534628-supplementary.pdf]

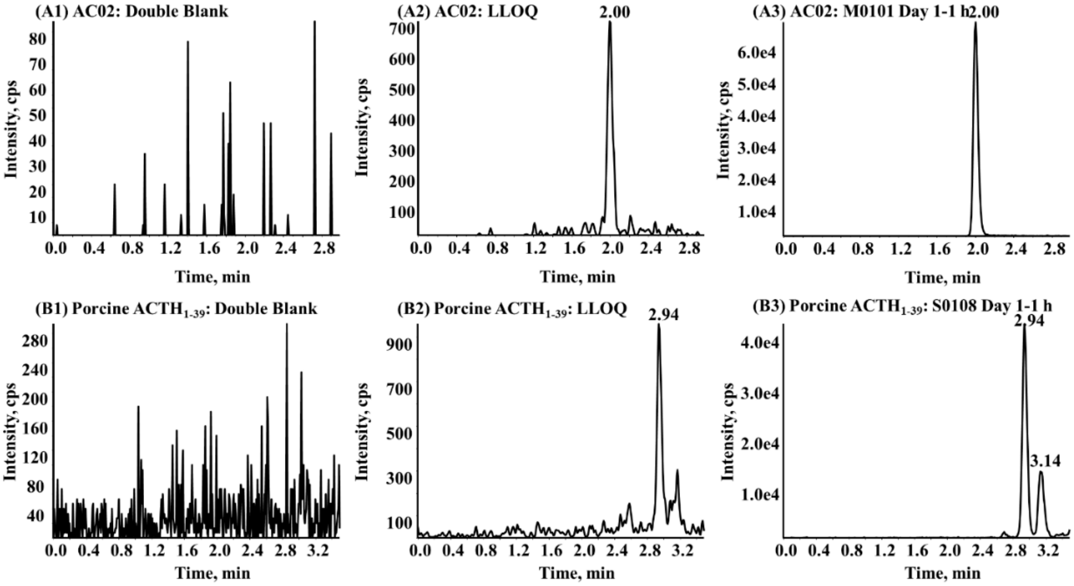

Figure S1. Representative MRM chromatograms of AC02 (A1: blank sample, A2: LLOQ sample of 0.500 ng/mL AC02, A3: study sample of 45.3 ng/mL), and porcine $ACTH_{1-39}$ (B1: blank sample, B2: LLOQ sample of 0.100 ng/mL porcine $ACTH_{1-39}$, B3: study sample of 3.55 ng/mL).

(A) AC02 lipemic sample

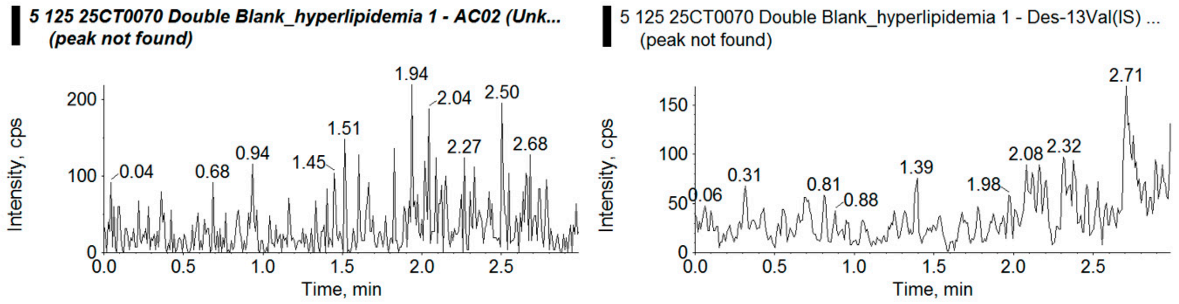

(B) AC02 hemolyzed sample

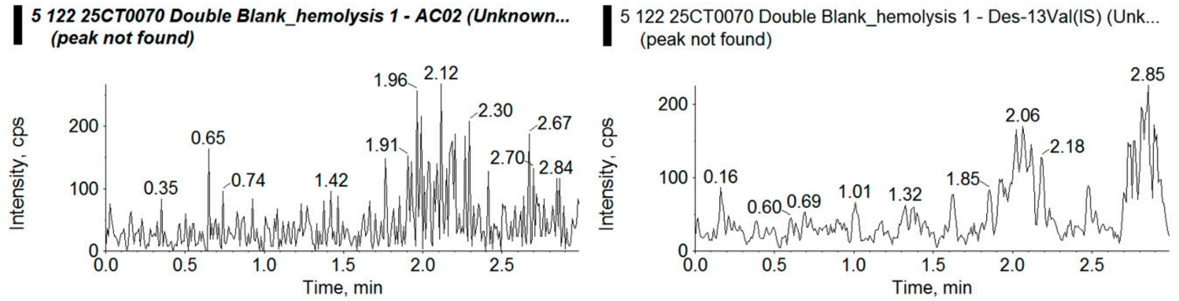

(C) Porcine ACTH$_{1-39}$ lipemic sample

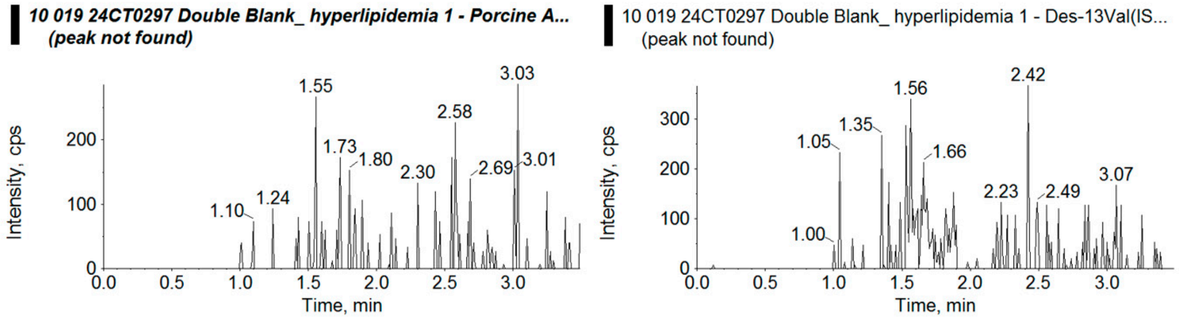

(D) Porcine ACTH$_{1-39}$ hemolyzed sample

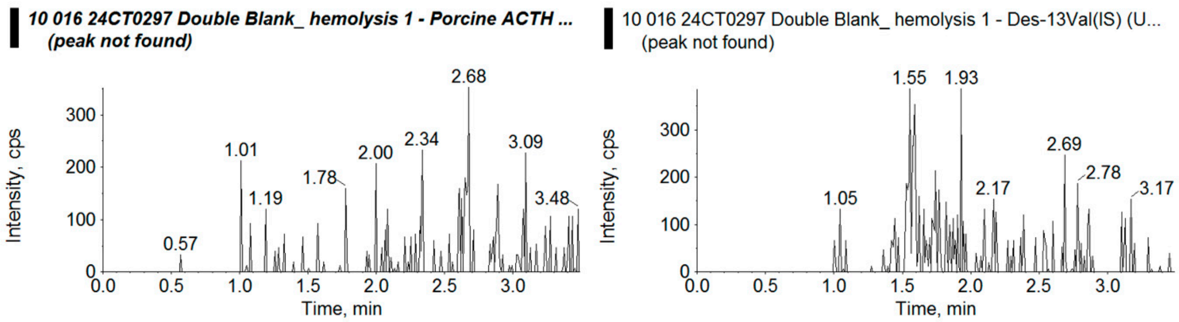

Figure S2. Representative hemolyzed and lipemic matrix chromatograms of AC02 (A: lipemic sample, B: hemolyzed sample; left: AC02, right: des-val^{13}), and porcine ACTH$_{1-39}$ (C: lipemic sample, D: hemolyzed sample; left: porcine ACTH$_{1-39}$, right: des-val^{13}).

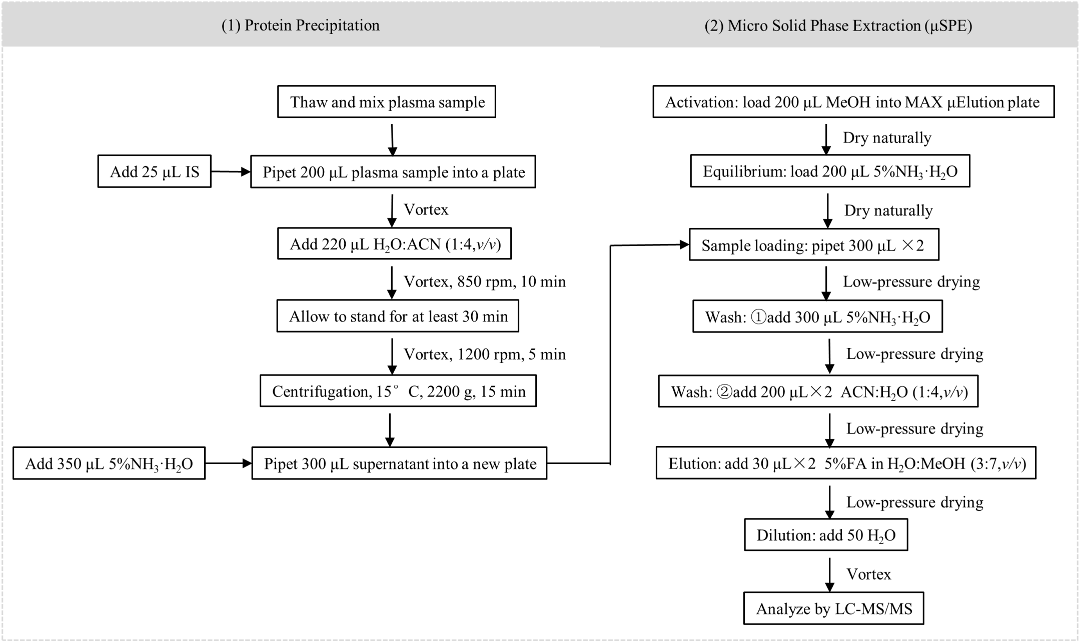

Figure S3. Workflow of the sample preparation protocol for porcine $ACTH_{1\text{-}39}$. The procedure consisted of two steps: (1) protein precipitation and (2) micro solid phase extraction (μSPE).

Table S1. Selection of precursor ion charge state

Analyte	Precursor ion charge	Parent ion	Product ion	Intensity	Selection
AC02	+7	649.7	-	-	×
	+6	757.5	876.0	3.7e7	√
	+5	909.3	973.2	9e7	×
			1010.0	8e7	×
	+4	1136.1	1346.0	2.8e8	×
Des-Val[13]	+7	635.5	-	-	×
	+6	741.2	856.2	2.4e7	√
	+5	889.3	948.3	5.1e7	×
			985.2	4.5e7	×
	+4	1111.3	1313.0	1.3e8	×
Porcine $ACTH_{1\text{-}39}$	+7	653.4	729.7	-	×
	+6	762.0	881.1	1.6e8	√
	+5	914.1	979.4	1.2e8	×
			1016.2	1e8	×
	+4	1142.4	1354.6	1.4e8	×
Human $ACTH_{1\text{-}39}$	+7	649.5	808.2	-	×
	+6	757.6	876.0	2.2e7	√
	+5	908.8	972.9	9e7	×
			1009.7	8e7	×
	+4	1135.7	1345.7	2.2e8	×

"–" indicates that the signal intensity was too low and not required for the comparison.

Table S2. Calibration curves of AC02

Run ID	0.500 ng/mL	Bias(%)	1.00 ng/mL	Bias(%)	4.00 ng/mL	Bias(%)	15.0 ng/mL	Bias(%)	60.0 ng/mL	Bias(%)	120 ng/mL	Bias(%)	160 ng/mL	Bias(%)	200 ng/mL	Bias(%)	Calibration curve
1	0.474	-5.2	0.952	-4.8	3.75	-6.3	15.5	3.3	65.9	9.8	108	-10.0	160	0.0	190	-5.0	y=0.05338x+0.003383
	0.529	5.8	1.04	4.0	4.05	1.3	15.8	5.3	61.3	2.2	120	0.0	166	3.8	190	-5.0	, R^2=0.9961
2	0.542	8.4	0.912	-8.8	4.15	3.8	15.3	2.0	58.0	-3.3	119	-0.8	150	-6.3	184	-8.0	y=0.05320x+0.003373
	0.472	-5.6	1.01	1.0	4.18	4.5	15.8	5.3	62.2	3.7	121	0.8	163	1.9	204	2.0	, R^2=0.9967
3	0.408	-18.4	*1.20	20.0	4.14	3.5	14.9	-0.7	63.5	5.8	120	0.0	170	6.3	200	0.0	y=0.05613x+0.003351
	0.588	17.6	0.995	-0.5	4.25	6.3	14.6	-2.7	58.5	-2.5	114	-5.0	158	-1.3	184	-8.0	, R^2=0.9925

Note: Each run contains two calibration curves. Regression equation: y = ax + b, weighting factor: $1/x^2$. *Represents the corresponding bias was out of the acceptance criteria.

Table S3. Calibration curves of porcine $ACTH_{1\text{-}39}$

Run ID	0.100 ng/mL	Bias(%)	0.200 ng/mL	Bias(%)	0.400 ng/mL	Bias(%)	1.20 ng/mL	Bias(%)	3.00 ng/mL	Bias(%)	6.00 ng/mL	Bias(%)	8.00 ng/mL	Bias(%)	10.0 ng/mL	Bias(%)	Calibration curve
1	0.0916	-8.4	0.210	5.0	0.392	-2.0	1.2	0.0	3.13	4.3	5.55	-7.5	7.83	-2.1	10.1	1.0	y=0.0002863x+0.003277, R^2=0.9970
	0.103	3.0	0.217	8.5	0.392	-2.0	1.18	-1.7	2.98	-0.7	5.71	-4.8	8.24	3.0	10.4	4.0	
2	0.100	0.0	0.195	-2.5	0.382	-4.5	1.19	-0.8	2.95	-1.7	5.67	-5.5	7.83	-2.1	10.0	0.0	y=0.0002735x+0.009162, R^2=0.9984
	*0.0606	-39.4	0.204	2.0	0.414	3.5	1.21	0.8	3.00	0.0	6.24	4.0	7.89	-1.4	10.8	8.0	
3	0.0846	-15.4	0.219	9.5	0.407	1.8	1.17	-2.5	3.06	2.0	5.66	-5.7	8.04	0.5	9.50	-5.0	y=0.0002912x+0.004853, R^2=0.9945
	0.105	5.0	0.219	9.5	0.411	2.8	1.28	6.7	2.96	-1.3	5.84	-2.7	7.72	-3.5	9.92	-0.8	

Note: Each run contains two calibration curves. Regression equation: $y = ax + b$, weighting factor: $1/x^2$. *Represents the corresponding bias was out of the acceptance criteria.

Table S4. Intra- and inter-run accuracy and precision of AC02 and porcine $ACTH_{1-39}$

Analyte	Nominal conc. (ng/mL)	Intra-run (*n* = 6)			Inter-run (*n* = 18)		
		Mean ± SD (ng/mL)	Precision (RSD%)	Accuracy (RE%)	Mean ± SD (ng/mL)	Precision (RSD%)	Accuracy (RE%)
AC02	0.500	0.471 ± 0.0871	18.5	-5.8	0.502 ± 0.0738	14.7	0.4
	1.50	1.48 ± 0.144	9.7	-1.3	1.60 ± 0.141	8.8	6.7
	10.0	10.6 ± 0.625	6.0	6.0	10.5 ± 0.551	5.2	5.0
	80.0	81.9 ± 1.92	2.3	2.4	81.7 ± 2.46	3.0	2.1
	150	152 ± 6.89	2.4	1.3	154 ± 4.89	3.2	2.7
Porcine $ACTH_{1-39}$	0.100	0.106 ± 0.00865	8.2	6.0	0.0977 ± 0.0118	12.1	-2.3
	0.300	0.303 ± 0.0228	7.5	1.0	0.293 ± 0.0176	6.0	-2.3
	1.00	0.987 ± 0.0390	4.0	-1.3	0.993± 0.0367	3.7	-0.7
	4.00	3.85 ± 0.129	3.4	-3.8	3.93 ± 0.187	4.8	-1.8
	7.50	7.26 ± 0.173	2.4	-3.2	7.43 ± 0.386	5.2	-0.9

Abbreviation: n, number of replicates. SD, standard deviation. RSD, relative standard deviation. RE, relative error.

Table S5. Recovery and matrix effects of AC02 and porcine $ACTH_{1-39}$ in plasma

Analyte	Nominal conc. (ng/mL)	Recovery (*n* = 6)		Matrix Effect (*n* = 6)		
		Mean ± SD (%)	Precision (RSD%)	Mean ± SD (%)	Precision (RSD%)	Accuracy (RE%)
AC02	1.50	82.7 ± 2.81	3.4	1.48 ± 0.0898	6.1	-1.3
	80.0	80.6 ± 1.51	1.9	-	-	-
	150	78.9 ± 2.17	2.8	151 ± 6.66	4.4	0.7
Porcine $ACTH_{1-39}$	0.300	83.8 ± 4.70	5.6	0.302 ± 0.0244	8.1	0.7
	4.00	89.3 ± 1.41	1.6	-	-	-
	7.50	90.5 ± 1.40	1.5	7.18 ± 0.205	2.9	-4.3

Abbreviation: n, number of replicates. SD, standard deviation. RSD, relative standard deviation. RE, relative error.

Table S6. The matrix stability of AC02 and porcine $ACTH_{1-39}$ (n = 3).

Analyte	Type of stability	Stability conditions	RSD (%)		RE (%)	
			LQC	HQC	LQC	HQC
AC02	Freeze-thaw	-20℃, five cycles	5.6	2.1	-2.7	-0.7
		-70℃, five cycles	5.0	3.4	-4.0	0.7
	Benchtop	RT, 2.4 h	5.6	1.9	-4.7	0.0
		Ice bath, 20.2 h	6.9	2.7	3.3	-2.0
	Long-term	-20℃, 78 d	5.0	1.6	-0.7	6.7
		-70℃, 78 d	12.5	1.9	-2.7	7.3
	Autosampler	8℃, 3 d 8 h	11.5	2.9	-3.3	6
	whole blood	Ice bath, 1 h	8.2	6.7	3.2	14.3
		Ice bath, 2 h	6.9	6.9	1.5	1.5
Porcine $ACTH_{1-39}$	Freeze-thaw	-20℃, five cycles	5.6	1.3	2.0	-2.0
		-70℃, five cycles	10.4	0.8	2.0	-4.5
	Benchtop	RT, 2.5 h	2.3	0.6	5.3	0.5
		Ice bath, 19.8 h	3.0	0.3	3.7	0.1
	Long-term	-20℃, 61 d	3.1	3.1	-4.3	-4.5
		-70℃, 61 d	2.4	3.8	-1.3	-4.1
	Autosampler	8℃, 2 d 4 h	6.1	2.1	-4.0	-6.4
	whole blood	Ice bath, 1 h	10.7	9.2	3.8	2.1
		Ice bath, 2 h	3.3	3.6	0.0	-0.5

Abbreviation: RSD, relative standard deviation. RE, relative error.

Table S7. The pharmacokinetic parameters of AC02 and porcine $ACTH_{1-39}$

PK Parameters (unit)	Mean ± SD (CV%) (n = 10)		
	AC02 (0.04 mg/kg)	AC02 (0.08 mg/kg)	Porcine $ACTH_{1-39}$ (25 U)
T_{max} (h)*	2.00 (1.00, 2.08)	2.00 (0.50, 2.08)	1.00 (0.50, 2.00)
C_{max} (ng/mL)	76.88 ± 26.51 (34.5%)	140.84 ± 46.80 (33.2%)	3.99 ± 0.59 (14.7%)
AUC_{0-t} (h·ng/mL)	115.10 ± 20.42 (17.7%)	217.28 ± 31.00 (14.3%)	6.83 ± 1.01 (14.8%)
AUC_{inf} (h·ng/mL)	115.59 ± 20.36 (17.6%)	217.49 ± 30.92 (14.2%)	6.88 ± 1.04 (15.1%)
$t_{1/2}$ (h)	0.11 ± 0.01 (9.1%)	0.12 ± 0.02 (12.5%)	0.14 ± 0.02 (15.3%)

Abbreviation: n, number of replicates. SD, standard deviation. CV, coefficient of variation. *T_{max} is expressed as the median (min, max).
